# Changes in Youth Cannabis Use After an Increase in Cannabis Minimum Legal Age in Quebec, Canada

**DOI:** 10.1001/jamanetworkopen.2022.17648

**Published:** 2022-06-17

**Authors:** Hai V. Nguyen, Shweta Mital

**Affiliations:** 1 School of Pharmacy, Memorial University of Newfoundland, St John’s, Canada

## Abstract

**Importance:**

In January 2020, Quebec raised the minimum legal age (MLA) for cannabis from 18 to 21 years. Evidence is needed to inform the ongoing debate on this policy. Although proponents believe that a higher MLA will protect youths from the harms of cannabis use, critics argue that it will push them back to the illegal market.

**Objective:**

To investigate changes in youth cannabis use after an increase in MLA for cannabis in Quebec.

**Design, Setting, and Participants:**

This cross-sectional study with difference-in-differences analysis compared changes in cannabis use among youths aged 15 to 20 years in Quebec vs all other Canadian provinces before and after Quebec’s increase in MLA. All estimates in descriptive and regression analyses were weighted. Nationally representative data from the National Cannabis Surveys 2018-2020 were used.

**Intervention:**

Increase in MLA for cannabis in Quebec implemented in January 2020.

**Main Outcomes and Measures:**

Past-3-month cannabis use.

**Results:**

The study sample included 1005 respondents (mean [SD] age, 17.5 [1.7] years; 50.2% [SD, 50.0%] male). After policy implementation, the increase in past-3-month cannabis use among youths aged 18 to 20 was 16.4 percentage points (95% CI, −27.3 to −5.5 percentage points; *P* = .01), or 51%, lower in Quebec than in other provinces. Meanwhile, no significant change in cannabis use among youths aged 15 to 17 years was found. The results were robust to several checks, including accounting for possible confounding effects of the COVID-19 pandemic on cannabis use.

**Conclusions and Relevance:**

In this study, an increase in the MLA from 18 to 21 years in Quebec was associated with a significantly lower increase in cannabis use among youths aged 18 to 20 years but no change in cannabis use among those aged 15 to 17 years. These findings can help to alleviate concerns that youths would switch to illegal markets in response to a higher MLA.

## Key Points

**Question:**

Is there an association of the increase in cannabis minimum legal age (MLA) from 18 to 21 years in Quebec, Canada, with youth cannabis use?

**Findings:**

In this cross-sectional study with difference-in-differences analysis of 1005 youths aged 15 to 20 years, although youth cannabis use still increased after the policy change, the increase in past-3-month cannabis use among youths aged 18 to 20 years was 51% lower in Quebec than in other provinces. There was no change in cannabis use among youths aged 15 to 17 years.

**Meaning:**

The lower increase in cannabis use among individuals aged 18 to 20 years alleviates concerns that youths would switch to illegal markets in response to a higher MLA; however, the increase in youth cannabis use despite the higher MLA highlights the need for additional policy measures to address rising youth cannabis use.

## Teaser

This difference-in-differences study uses data from the National Cannabis Survey to evaluate changes in youth cannabis use after Quebec raised the minimum legal age from 18 to 21 years.

## Introduction

Canada legalized recreational cannabis use in October 2018. The legalization was accompanied by a minimum legal age (MLA) law to prevent cannabis use among underaged youths. At the time of legalization, this MLA was set at 19 years in all provinces except Alberta and Quebec, which chose 18 years. Although the choice of a low MLA, such as 18 or 19 years, was supported by the need to divert youths from illegal to safer and regulated cannabis and to harmonize the MLA for cannabis with that for other substances,^1^ the majority of Canadians believed that the MLA for cannabis should be higher.^2^ For comparison, all US states that legalized cannabis have adopted an MLA of 21 years.^3^

In January 2020, Quebec raised the MLA for cannabis from 18 to 21 years and thus extended the ban on recreational cannabis to youths aged 18 to 20 years. This policy change aims to protect youths from the adverse effects of cannabis on developing brains.^4^ The policy also aligns with recommendations made by the Canadian Medical Association before the legalization on the basis of adverse cardiovascular, pulmonary, and neuropsychological effects of cannabis use.^5^ However, critics argue that this policy could undermine the purpose of cannabis legalization by inducing youths to go back to more harmful, illegal cannabis.^4^

Although the MLA increase from 18 to 21 years targets the 18- to 20-year age group, it may also affect those younger than 18. Evidence from Tobacco 21 laws in the US (which increased the MLA for cigarettes from 18 to 21 years) indicated that cigarette use declined among youths aged 16 to 17 years, who often realy on older peers to access tobacco products.^6^ It is also possible that a higher MLA sends a stronger message about the harms of cannabis use at young ages and hence further discourages its use among adolescents.

Despite the considerable policy interest and ongoing debate, evidence on the effects of raising the current MLA for cannabis is lacking. A previous study assessed the merits of alternative ages as the MLA by evaluating later-life outcomes of starting cannabis at different ages.^7^ However, that study examined only the association between different ages of first cannabis use and subsequent outcomes and used only prelegalization data.

In the current study, we provide the first evidence, to our knowledge, on the association between an increase in MLA for cannabis from 18 to 21 years and youth cannabis use by studying the experience of the Canadian province of Quebec and using the remaining 9 provinces that did not adopt a higher MLA as a reference group. We examine the changes among all youths aged 15 to 20 years and separately for ages 15 to 17 years and 18 to 20 years. Findings from our study will be useful not only to inform the ongoing debate on the optimal MLA for cannabis use in Canada but also to guide policy choices of MLA for cannabis use in other jurisdictions that are considering recreational cannabis legalization.

## Methods

Because this cross-sectional study with a difference-in-differences (DD) analysis used deidentified secondary data, ethics approval was not required based on Newfoundland and Labrador’s Health Research Ethics Board guidelines. This study followed the Strengthening the Reporting of Observational Studies in Epidemiology (STROBE) reporting guideline.

### Data Source and Study Outcome

We used individual-level data from 6 waves of the National Cannabis Survey (NCS).^8^ The NCS is a nationally representative cross-sectional survey of cannabis use among Canadians aged 15 years or older. These quarterly surveys interview more than 5000 respondents with the use of electronic questionnaires (either online or via computer-assisted telephone).^8^ The survey uses a 2-stage stratified sampling design in which households are sampled for each province in the first stage and individuals are sampled in the second stage.^8^ The response rates for the survey waves included in our study ranged from 46% to 51%.^8^ The NCS is particularly suited for this analysis because it has collected data on cannabis use both before and after the MLA increase in Quebec.

The outcome of interest was an indicator of past-3-month cannabis use. This outcome captured whether a respondent used cannabis at least once in the past 3 months based on the following survey question: “During the past 3 months, how often did you use cannabis?: not in the past 3 months, once or twice, monthly, weekly, daily, almost daily.” In a robustness check, we also examined past-3-month cannabis initiation as an outcome. This outcome captured whether a respondent started using cannabis in the preceding 3 months and was based on the following survey question: “Did you start using cannabis in the past 3 months?: yes; no, I started more than 3 months ago.”

### Study Sample and Study Period

We used NCS data for 1005 youths aged 15 to 20 years that spanned the period from quarter 4 of 2018 to quarter 4 of 2020. We chose quarter 4 of 2018 as the starting point because recreational cannabis was legalized in Canada in 2018 and all provinces first set 18 or 19 years as the MLA in this quarter. Quarter 4 of 2020 was the latest available NCS cycle. We note that although the NCS was conducted quarterly between quarter 4 of 2018 and quarter 4 of 2019, it was paused during quarter 1 of 2020 to quarter 3 of 2020 because of the COVID-19 pandemic. Thus, in this analysis, we used data from 5 survey cycles conducted in the 5 quarters immediately preceding Quebec’s MLA increase policy (ie, quarter 4 of 2018 to quarter 4 of 2019) and 1 survey cycle conducted approximately 1 year after the policy (ie, quarter 4 of 2020).

### Statistical Analysis

We used the quasi-experimental DD method^9,10,11,12,13^ to compare before-and-after changes in past-3-month cannabis use in Quebec (where the MLA was increased to 21 years) with similar changes in other Canadian provinces (where the MLA remained at 18 or 19 years). We estimated DD regressions using individual-level data. The covariate of interest was an indicator for the MLA increase policy, which was equal to 1 if the respondent resided in Quebec after January 2020 and 0 otherwise. The regression analyses controlled for respondent age (measured as a continuous variable), male sex (nonmale sex was the reference category), and household size (measured as a continuous variable). In addition, these regressions included province indicators to control for all time-invariant characteristics of provinces and quarter-year indicators to control for secular changes or shocks in outcomes that were common to Quebec and other provinces.

We estimated the DD regressions for the full sample and separately for the samples of adolescents (ages 15-17 years) and young adults (ages 18-20 years). We modeled all outcomes using linear probability regressions to produce unbiased estimates in fixed-effects analyses^14^ and for ease of interpreting the marginal effects.^15,16^ All estimates in descriptive and regression analyses were weighted. Standard errors were clustered at the province level to account for within-cluster correlation and serial correlation over time.^17^ Analyses were performed using Stata, version 16 software (StataCorp LLC).^18^ Tests were 2-sided, and a 5% significance level was used.

We conducted several analyses to investigate the robustness of our results. First, we addressed the possibility that any observed changes in youth cannabis use after the MLA increase could be driven by differences in ease of access to cannabis over time between Quebec and the other provinces rather than by Quebec’s higher MLA policy. To do so, we controlled for the number of cannabis retail stores per 100 000 population at the province-quarter level. Second, because Quebec’s higher MLA was implemented just before the COVID-19 pandemic began, our estimate of the changes in cannabis use after the MLA increase may additionally capture differences in effects of the pandemic on cannabis use in Quebec vs the other provinces. To address this potential confounding, we reestimated the DD regressions but compared Quebec with only non-Atlantic provinces (Ontario, Manitoba, Saskatchewan, Alberta, and British Columbia). These non-Atlantic provinces, which were relatively more affected by the COVID-19 pandemic than the Atlantic provinces (Newfoundland and Labrador, Nova Scotia, New Brunswick, and Prince Edward Island), were more comparable with Quebec in terms of the pandemic experience.^19^ Third, we reestimated the DD regressions by dropping different reference provinces one at a time. This analysis informed whether our findings were influenced by any individual reference province. Fourth, we examined the sensitivity of our results to the inclusion of respondents’ household income as a control; we did not control for respondents’ household income in the main analysis because more than 10% of respondents did not report this information. Finally, we examined changes in the likelihood of past-3-month cannabis initiation after Quebec increased the MLA. If the direction of any change in past-3-month initiation was the same as that of past-3-month cannabis use, our base case findings would be strengthened.

## Results

### Descriptive Statistics

The study sample included 1005 respondents (134 in Quebec and 871 in the reference group of all other Canadian provinces). In the full sample, the mean (SD) age of respondents was 17.5 (1.7) years; 50.2% (SD, 50.0%) of the sample was male, and 49.8% (SD, 50.0%) was nonmale (including female and gender diverse); and respondents had 4.1 (SD, 1.3) household members on average (Table). The NCS does not collect data on race and ethnicity. Quebec (the treated province) was similar to the reference provinces in terms of the respondents’ age (mean [SD], 17.4 [1.6] years vs 17.5 [1.7] years) and household size (mean [SD], 3.9 [1.1] members vs 4.2 [1.3] members); however, it had a higher proportion of male respondents than the reference group (mean, 58.2% [SD, 49.5%] vs 48.2% [SD, 50.0%]).

**Table.** Descriptive Statistics^a^

Characteristic	Mean (SD)		
	Full sample (N = 1005)	Quebec (n = 134)	Reference group^b^ (n = 871)
Age, y	17.5 (1.7)	17.4 (1.6)	17.5 (1.7)
Sex, %			
Male	50.2 (50.0)	58.2 (49.5)	48.2 (50.0)
Nonmale^c^	49.8 (50.0)	41.8 (49.3)	51.8 (50.0)
Household size, No.	4.1 (1.3)	3.9 (1.1)	4.2 (1.3)

^a^ Data are from the National Cannabis Survey quarter 4 of 2018 to quarter 4 of 2020. Weighted percentages for indicator variables and weighted means for continuous variables are presented. The sample includes all respondents aged 15 to 20 years.

^b^ Reference group includes Nova Scotia, Ontario, Prince Edward Island, New Brunswick, Newfoundland and Labrador, Manitoba, British Columbia, Alberta, and Saskatchewan.

^c^ Nonmale sex includes female and gender diverse respondents. The proportion of gender diverse respondents was too small to be reported separately while maintaining respondent anonymity.

**Figure 1.**
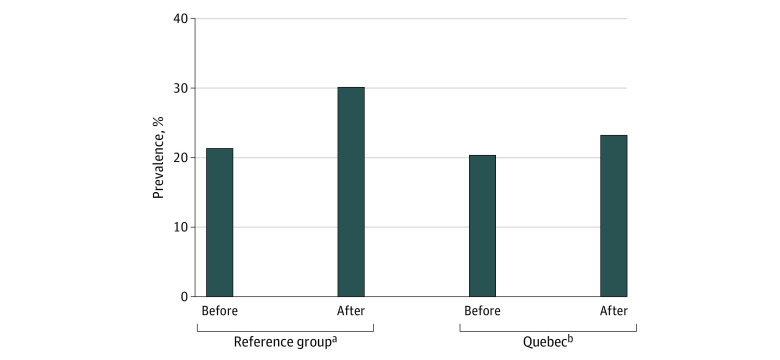
Unadjusted Cannabis Use Before and After Quebec’s Minimum Legal Age Increase vs the Reference Group Data are from the National Cannabis Survey quarter 4 of 2018 to quarter 4 of 2020. The reference group includes Nova Scotia, Ontario, Prince Edward Island, New Brunswick, Newfoundland and Labrador, Manitoba, British Columbia, Alberta, and Saskatchewan. ^a^*P* = .13. ^b^*P* = .79.

Figure 1 shows a comparison of cannabis use before and after the MLA increase. Although respondents in both Quebec and the reference group reported increases in cannabis use after Quebec’s MLA increase, the increase in Quebec (from 20.4% to 23.3%) was lower than the increase in the reference group (from 21.4% to 30.1%).

### Regression Estimates

**Figure 2.**
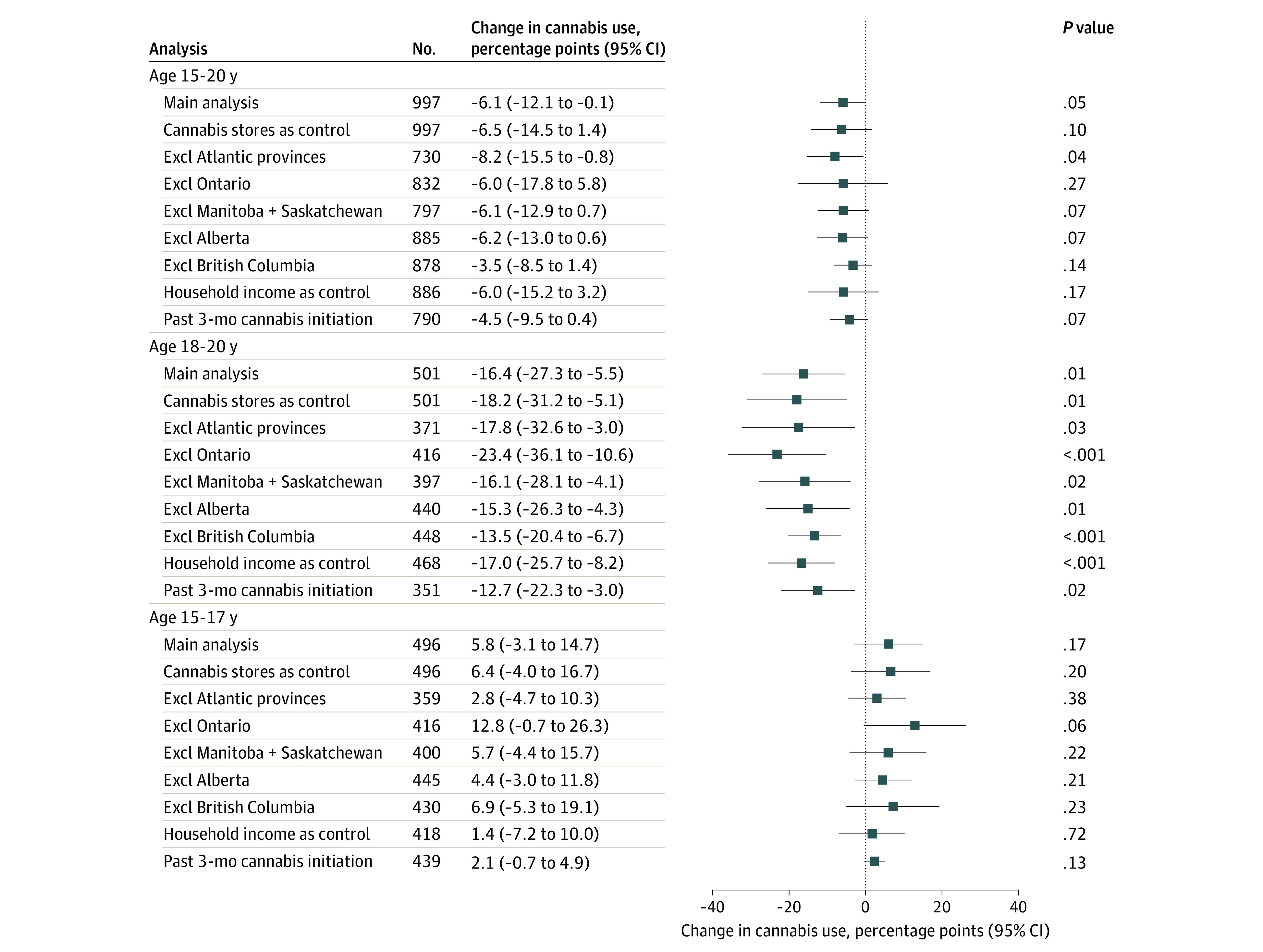
Quebec’s Higher Cannabis Minimum Legal Age (MLA) and Youth Cannabis Use: Difference-in-Differences Estimates Data are from the National Cannabis Survey quarter 4 of 2018 to quarter 4 of 2020. Estimates shown as percentage point changes for the main analysis and each robustness check. Each estimate is from a separate regression model. All regressions were estimated using ordinary least squares and included province and year as fixed effects while controlling for age, sex (male; nonmale is the excluded category), and household size. Standard errors are clustered at the province level. All estimates are weighted. Atlantic provinces include Newfoundland and Labrador, Nova Scotia, New Brunswick, and Prince Edward Island. Excl indicates excluding.

The regression results are shown in Figure 2 for the full sample and separately for youths aged 18 to 20 and 15 to 17 years. In the full sample, the increase in cannabis use after the MLA increase in Quebec was 6.1 percentage points (95% CI, 12.1 to 0.1 percentage points; *P* = .047) lower than the corresponding increase in the reference provinces. Compared with the 20.4% prepolicy prevalence of cannabis use in Quebec, this decline means that the increase in youths’ cannabis use in Quebec was 30% lower than in other provinces after the increase in MLA in Quebec. Analyses by age group indicated that the relative decline in youths’ cannabis use was driven by the change in the 18 to 20 age group. Specifically, cannabis use among 18- to 20-year-olds in Quebec was 16.4 percentage points (95% CI, 27.3 to 5.5 percentage points; *P* = .01) lower relative to the corresponding change in the reference provinces (or a 51% decline relative to the prepolicy prevalence of 31.9% in this age group). Meanwhile, we observed no significant change in cannabis use among those aged 15 to 17 years after the MLA increase (5.8 percentage points; 95% CI, −3.1 to 14.7; *P* = .17).

Our results were robust to several additional analyses (Figure 2). Our finding of a smaller increase in cannabis use in the 18- to 20-year age group continued to hold when we additionally controlled for the quarterly number of cannabis retail stores in each province (−18.2 percentage points; 95% CI, −31.2 to −5.1 percentage points; *P* = .01). The policy coefficients remained large and significant when we excluded the Atlantic provinces from the analysis (−17.8 percentage points; 95% CI, −32.6 to −3.0 percentage points; *P* = .03) and when we excluded the other reference provinces, one at a time (Ontario, −23.4 percentage points [95% CI, −36.1 to −10.6 percentage points; *P* < .01]; Manitoba and Saskatchewan, −16.1 percentage points [95% CI, −28.1 to −4.1 percentage points; *P* = .02]; Alberta, −15.3 percentage points [95% CI, −26.3 to −4.3 percentage points; *P* = .01]; British Columbia, −13.5 percentage points [95% CI, −20.4 to −6.7 percentage points; *P* < .01]). Our results were also similar to the main analysis when we controlled for respondents’ household income (−17.0 percentage points; 95% CI, −25.7 to −8.2 percentage points; *P* < .01). Furthermore, we found that the MLA increase in Quebec was associated with lower cannabis initiation in the past 3 months among youths aged 18 to 20 years (−12.7 percentage points; 95% CI, −22.3 to −3.0 percentage points; *P* = .02) relative to those in the reference provinces. This finding lends additional support to that of a slower increase in cannabis use among youths aged 18 to 20 years after the MLA increase in Quebec.

Our finding of no change in cannabis use for the 15- to 17-year age group was also robust to the different checks. The changes in cannabis use remained insignificant even when we controlled for the number of cannabis retail stores, excluded subsets of reference provinces, and controlled for household income (Figure 2).

## Discussion

This study provides, to our knowledge, the first evidence on the association between an increase in MLA for recreational cannabis from 18 to 21 years in Quebec, Canada, and youths’ cannabis use. We found that although cannabis use increased among youths in all provinces even after Quebec raised the MLA, the increase among youths aged 18 to 20 years was 16.4 percentage points, or 51%, lower in Quebec relative to the other provinces. Meanwhile, the higher MLA was not associated with changes in cannabis use among youths aged 15 to 17 years.

The smaller increase in cannabis use among youths aged 18 to 20 years after Quebec raised the MLA can help to address the concern that such a policy may cause youths to switch back to illegal markets. Although we were unable to directly evaluate changes in youths’ cannabis purchases from the illegal market, the NCS question captured all cannabis use (including that from illegal markets). Thus, the large net reduction in cannabis use that we found suggests that any increase in illegal cannabis use was far exceeded by the reduction in legal cannabis use. From a public health perspective, the large reduction in cannabis use among youths aged 18 to 20 years suggests that the higher MLA policy could lead to substantial public health benefits, especially because prior to the increase in MLA in 2020, more than one-quarter of all cannabis users aged 15 to 20 years initiated cannabis between the ages of 18 and 20 years.^20^

Previous studies have documented seasonality in patterns of cannabis use. Specifically, cannabis use is lowest in the first quarter and highest in the last quarter of the year.^21^ In our analysis, we compared cannabis use from quarter 4 of 2020 after Quebec’s MLA increase with that in quarters before the MLA increase. It will be of interest to investigate whether the increases in cannabis use in Quebec are even smaller when data for more quarters after the MLA increase become available. However, we note that if the seasonality effects are similar between Quebec and the other provinces, they would likely be cancelled out in our DD analyses.

Our results are consistent with evidence on raising the MLA for other substances to 21 years. Evidence from Tobacco 21 laws in the US suggests that raising the MLA for tobacco from 18 to 21 years reduces smoking and e-cigarette use among individuals aged 18 to 20 years.^6,22^ Similarly, an increase in the MLA for alcohol is associated with a reduction in youths’ alcohol consumption.^23^

We note, however, that the higher MLA was only associated with a slower rate of increase in cannabis use in Quebec compared with the other provinces; there was no absolute decrease in cannabis use among youths in Quebec after the province implemented the higher MLA. This increase, therefore, calls for additional policy measures, such as raising youth awareness on cannabis harms and school- and community-based cannabis use prevention programs to be adopted alongside the higher MLA to further reduce cannabis use among youths.

### Limitations

Our study has several limitations. First, we looked at only the 1-year changes after the MLA increase. Future research should examine whether these changes can be sustained and lead to better physical and mental health outcomes. Second, the period after policy implementation coincides with the COVID-19 pandemic, which affected Quebec and other provinces differently. However, our findings continued to hold in the robustness check, where we used the subset of reference provinces that was most similar to Quebec in its pandemic experience. Third, it is possible that youths switched to using other substances, such as tobacco, e-cigarettes, or alcohol, after the increase in MLA. Although we were unable to evaluate these substitution effects because the NCS does not collect information on other substance use, it is an important direction for future research. Furthermore, even though cannabis initiation among never-users aged 18 to 20 years was lower after the MLA increase, it will be interesting to evaluate whether the MLA increase also encouraged existing cannabis users in this age group to reduce the amount of cannabis consumed. Finally, there is evidence that adolescents who reside close to jurisdictional borders evade the MLA laws by purchasing restricted substances from neighboring jurisdictions where the MLA is lower.^24^ Thus, youths in Quebec could try to purchase recreational cannabis in the neighboring provinces of Ontario and New Brunswick, both of which have an MLA of 19. Although the available data did not allow us to test such evasion formally, our finding of a reduction in cannabis use in the 18 to 20 age group suggests that such evasion was low.

## Conclusions

In this study, the increase in Quebec’s cannabis MLA from 18 to 21 years was associated with a significantly lower increase in cannabis use among youths aged 18 to 20 years in Quebec than in other provinces. Although this finding helps to alleviate concerns that youths will switch to illegal markets in response to a higher MLA, the increase in youth cannabis use despite the higher MLA highlights the need for additional policy measures to address rising cannabis use among youths.
